# 3D Printing Improve the Effectiveness of Fracture Teaching and Medical Learning: A Comprehensive Scientometric Assessment and Future Perspectives

**DOI:** 10.3389/fphys.2021.726591

**Published:** 2021-12-24

**Authors:** Jian Shi, Shenao Fu, María José Cavagnaro, Shaokang Xu, Mingyi Zhao

**Affiliations:** ^1^Department of Spine Surgery, The Third Xiangya Hospital, Central South University, Changsha, China; ^2^Xiangya School of Medicine, Central South University, Changsha, China; ^3^College of Medicine-Phoenix, The University of Arizona, Phoenix, AZ, United States; ^4^Department of Pediatrics, The Third Xiangya Hospital, Central South University, Changsha, China

**Keywords:** 3D printing, teaching and learning, fracture, multidisciplinary cooperation, scientometric, advanced medical education

## Abstract

Fractures of complex body parts are often serious and difficult to handle, and they have high technical and training requirements. However, the realistic situation is that there are few opportunities for the junior residents, trainee doctors, and especially medical students to contact enough clinical practice and see such fracture patients. Fortunately, with the rapid development and continuous progress of 3D printing and related technologies, this situation has gradually gotten better and better. In this research, we confirmed that 3D printing technology could improve the effectiveness of fracture teaching and medical learning from multiple dimensions. We comprehensively screened and assessed 223 papers from the Web of Science (WoS) Core Collection on October 3, 2021, with “((3D) AND ((printing) OR (printed)) AND (fracture)) AND ((education) OR (training) OR (teaching))” as the retrieval strategy. Additionally, we used the VOSviewer software to analyze the keywords and countries and the organizations of the publications, then a series of scientometric and visualized analyses were made based on the retrieval results. Afterward, multiple databases were retrieved according to our selection criteria, we selected eight studies for the extensive literature analysis. The extracted data contained information of authors, problems solved, participants, methods, assessments, results, and benefits/limitations. These intuitive and in-depth analyses further confirmed and appraised the advantages of 3D printing in complex fracture models more objectively. In conclusion, 3D printing could improve the effectiveness and extension of fracture teaching, as well as medical learning, by providing the powerful interaction with 3D effect, wakening students learning interest, and allowing the junior residents, trainee doctors to have as realistic a virtual practice experience as possible. Through this research, it is expected that more researchers could be attracted to conduct more comprehensive and thorough studies on the application of 3D printing for training and educational propose, to promote the development of 3D technology-based medical education practice and further deepen the reform of medical education and improve the quality of fracture education and learning.

## Introduction

Fracture-associated education is a difficult part of the medical education system. It is a hard task for medical students to understand the fracture-associated basic anatomy and clinical knowledge well only with 2D models such as CT images, X-Ray images, etc. However, common 3D models and cadaveric specimens are usually normal types, and cadaveric specimens are not convenient to use for studying or reviewing all the time. Its rareness also causes limitation, so problems always exist. In recent years, 3D printing technology used in the medical field has become the hotspot, which can create 3D objects through successive deposition of materials in 2D layers. Furthermore, it has been used for surgery simulation, training and making teaching aids, etc. Hence, it is a potential solution for the problems mentioned above (Chae et al., 2015; Baskaran, 2016; Garcia et al., 2018; Oberoi et al., 2018; Pugliese et al., 2018). With the rapid development of 3D technology, more and more 3D printing and medical teaching or learning-related research have emerged. However, little research was carried out for the fundamental literature study about the application of 3D printing and fracture teaching with medical learning. Therefore, accurate evaluation and visualized analysis of relevant publications on the application of 3D printing in fracture education is particularly important.

Comprehensive scientometric assessment, empowered by visual or computational analytical approaches, provides unlimited possibilities to improve the accessibility, reproducibility, correctness, and timeliness of research on a specific category. Scientometric, as well as bibliometrics analysis, are all on the grounds of large-scale literature databases for deeply analyzing, which have become research hotspots and developing trends in lots of different fields (Sugimoto et al., 2019; Kagan et al., 2020; Chen et al., 2021; Shi et al., 2021). In this research, an essential analysis tool, VOSviewer (Van Eck and Waltman, 2017), developed by Nees Jan van Eck and Ludo Waltman at Leiden from Leiden University (Leiden, Netherlands) was used to perform the scientometric assessment and other analysis tasks. The purpose of this research was to select and evaluate current publications and analyze the effects on fracture-associated medical education of 3D printing. We also tried to make clear how efficient it could be when used in medical education. Additionally, after the scientometric assessment and prospect for the future, we hoped to clarify the future development direction of relative research.

## Materials and Methods

In this study, we searched the Web of Science (WoS) Core Collection on October 3, 2021, with the following retrieval strategy “((3D) AND ((printing) OR (printed)) AND (fracture)) AND ((education) OR (training) OR (teaching))”. In addition, the time frame was from 2000 to 2020, and we successfully gained 223 publications. Then, we used the VOSviewer software (Leiden, Netherlands), which excellently visualizes abstract concepts, to determine the co-occurrence analysis of all keywords. We also conducted the co-authorship of countries, and the co-author relationship of the organizations was analyzed. Afterward, the abstracts/titles of the outcomes were reviewed, and pieces of research on fracture-associated medical education were selected. Listed below are our selection criteria:

1) The research focuses on the effect of 3D printing on medical education.
2) The research conducted a rigorous comparative analysis experiment.
3) The research has a clear description of the assessment methods.
4) The test subjects of the research are medical undergraduates or residents.

We analyzed the following areas:

1) The problems the research solved.
2) The research participants.
3) The research methods.
4) The assessment methods.
5) The research results.
6) The benefits of 3D printing the research found.
7) The limitations of 3D printing that the research found.

## Results

**Figure 1:**
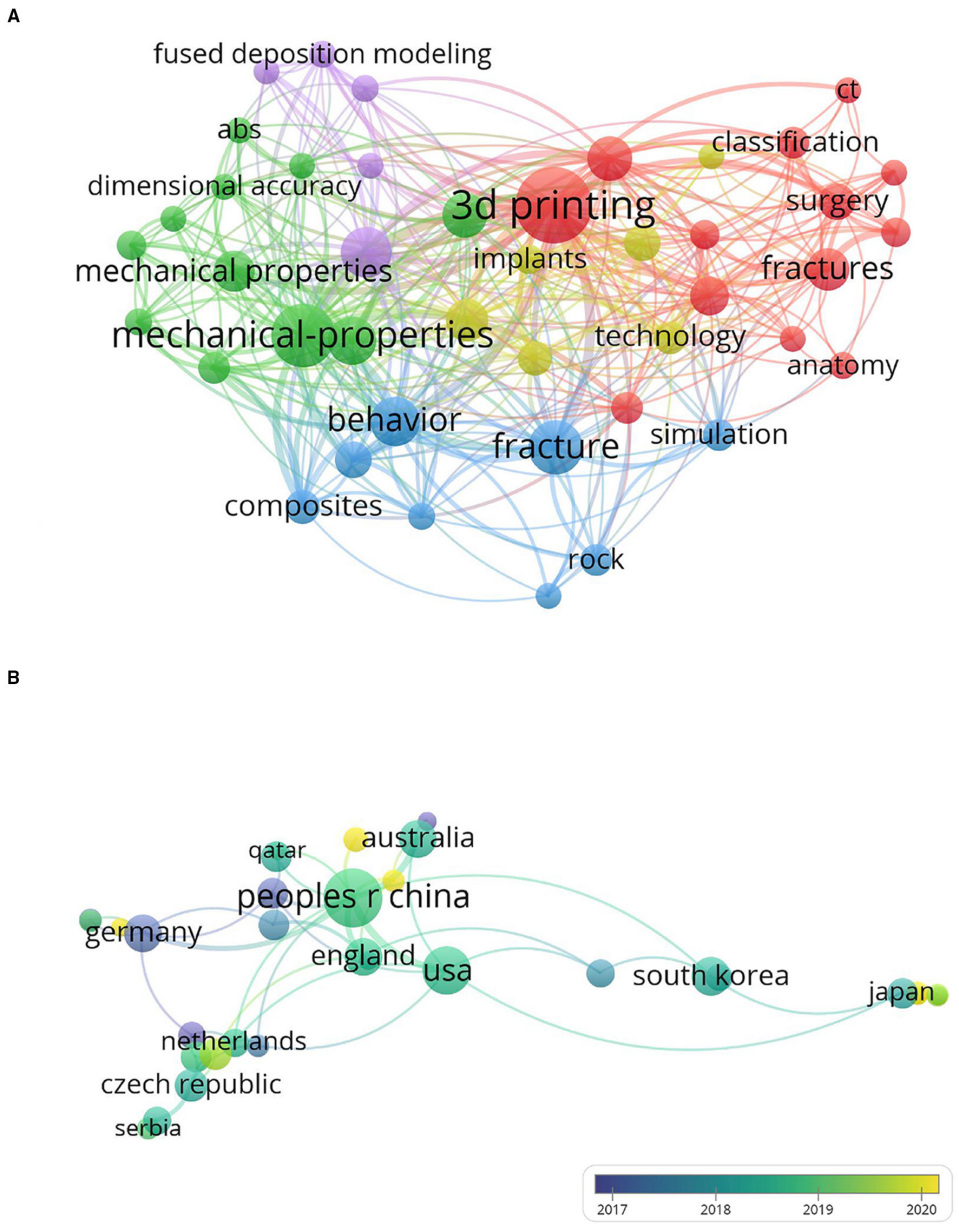
The visualization of keywords and co-author relationship of countries. **(A)** The co-occurrence network of keywords. **(B)** The image of the co-author relationship of countries.

A series of scientometric and visualized analyses were made based on the content retrieved from data sources. All results were summarized as follows: Figure 1A shows the common word network of the most common keywords in the target documents we studied. Research areas include teaching, design, prediction, treatment, and printing technology. In the past two decades, 3D printing has been increasingly linked to fractures. Among all keywords from the publications, 3D printing has the highest frequency and link strength. Besides, additive manufacturing, implants, mechanical properties, and fused deposition modeling also have multifarious to appear in different publications. We found that the articles from China, England, the USA, and Australia were mainly published in recent years while Germany, the Czech Republic, and Serbia have more previously published materials (Figure 1B).

**Figure 2:**
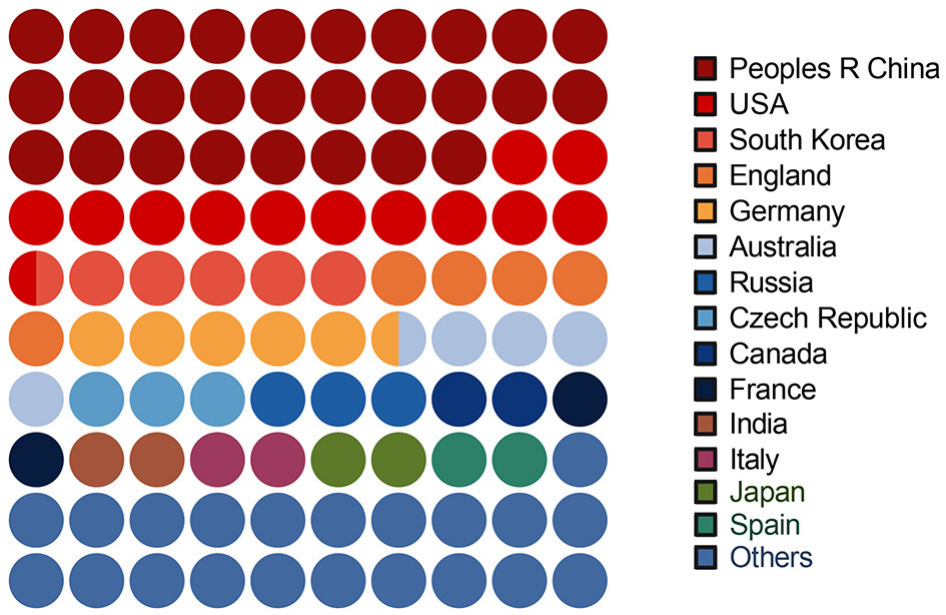
Country distribution for the articles.

We also found that most of the articles were published in China, the USA, South Korea, and England. However, the number of the other developing countries is relatively small. The two most productive countries, namely China and the USA, also have the most active partnership. Their main partners are Germany, England, Australia, and South Korea. Other main contributions to this field are from Russia, the Czech Republic, Canada, France, India, Italy, Japan, and Spain (Figure 2).

**Figure 3:**
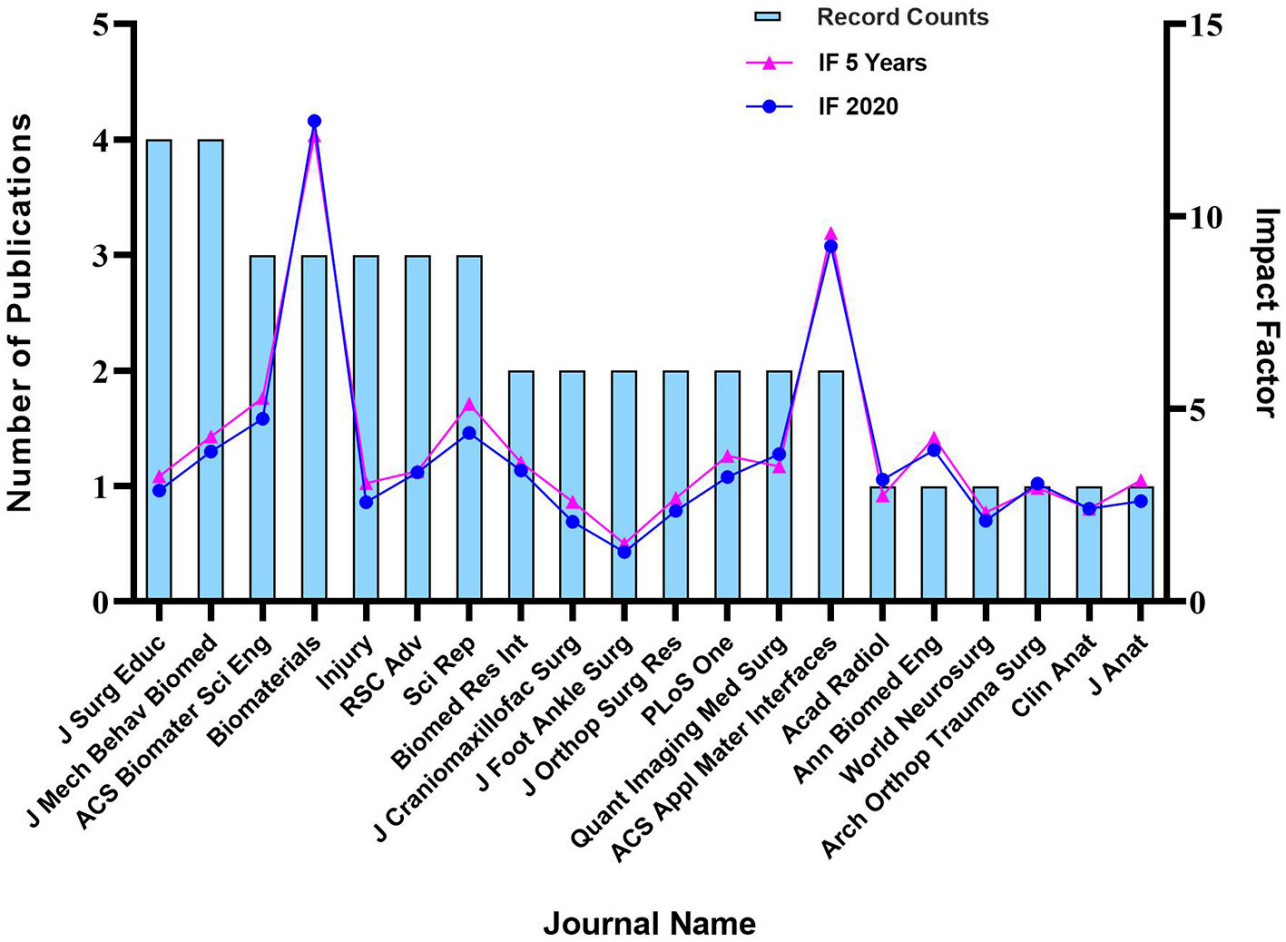
Impact factors and journal publication frequency.

**Figure 4:**
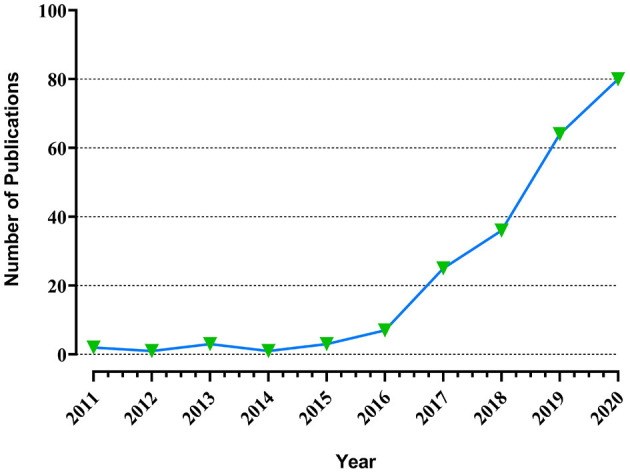
Year distribution of the selected articles.

**Figure 5:**
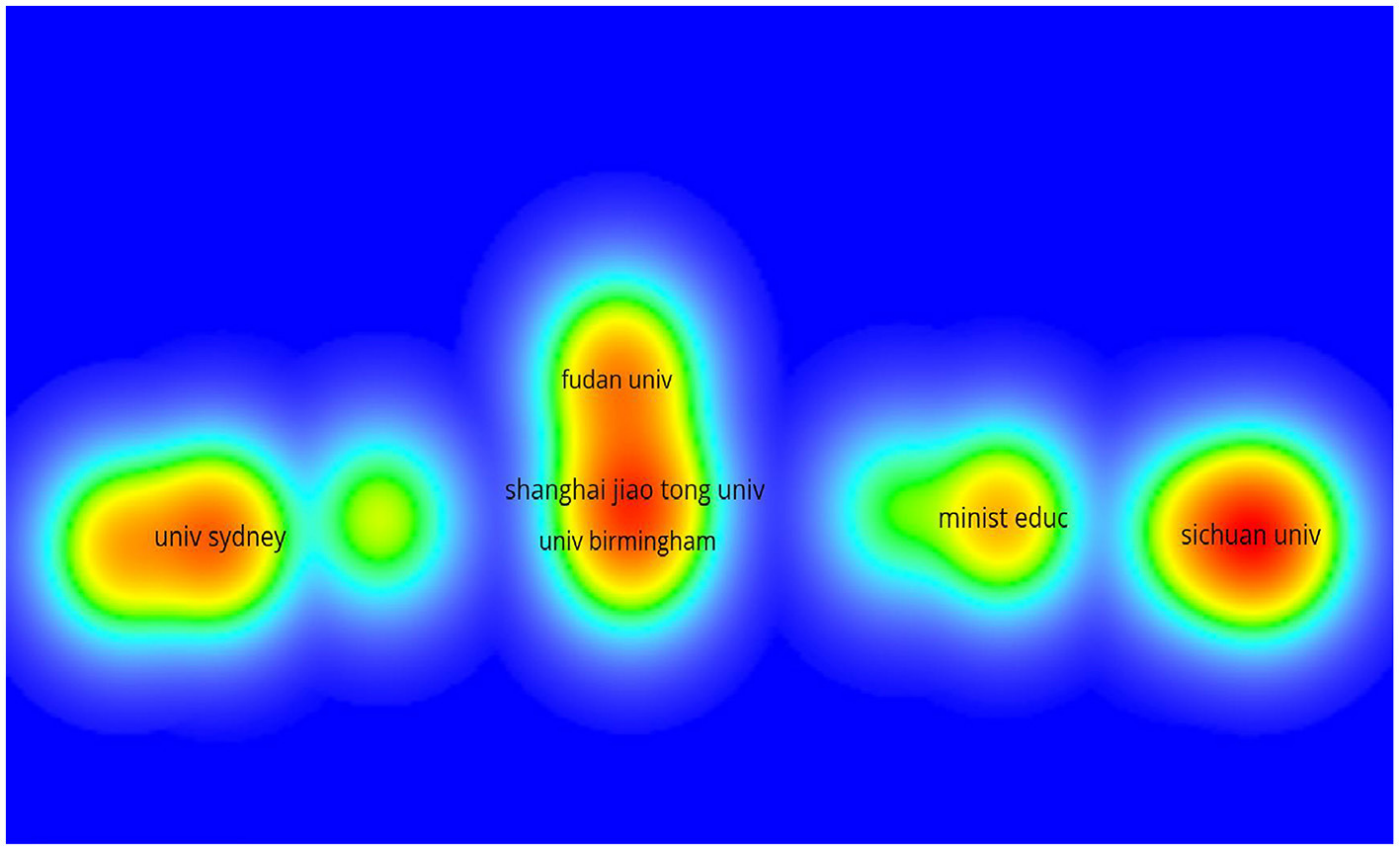
Visualization of number of publications issued by institutions and the density of co-author relationship.

Figure 3 enlists the journals of the articles in descending order by the impact factor and the average number of citations per paper (2020/5 years). Altogether, 20 journals were included with the leading impact factor of Biomaterials (IF: 12.479). Additionally, the distribution of selected per year was shown in Figure 4 while the number of publications issued by institutions and the density of co-author relationships were shown in Figure 5. We could clearly see the apparent upward trend of publications number year by year. Among all the organizations or institutions, Shanghai Jiao Tong University (China) and University of Sydney (Australia) are led by 6 publications. Other main contributions to this field are also from the University of Birmingham (England), Fudan University (China), and Sichuan University (China).

Afterward, we reviewed the titles and abstracts, excluding duplicates and getting a full text from multiple databases were retrieved according to our selection criteria, and eight studies were selected for the extensive literature analysis. The extracted data contained information of authors, problems solved, participants, methods, assessments, results, and benefits/limitations. In the screening process, non-medical or non-biological and veterinary-related publications were also not selected. Table 1 shows the comprehensive summaries of the selected studies (Li et al., 2015; Cao et al., 2017; Chuang et al., 2017; Huang et al., 2018; Lim et al., 2018; Meng et al., 2018; Wu et al., 2018; Tan et al., 2019).

**Table 1:** Summary table of published studies utilizing bone fracture with an assessment of the 3D printing.

**Authors**	**Problems solved**	**Participants**	**Methods**	**Assessments**	**Results**	**Benefits**	**Limitations**
Lim et al. (2018)	Acetabular fracture	41 orthopedic residents, PGY 1-5	Fifteen randomized testing stations with XR, CT scans, or 3D model of an acetabular fracture	Test and subjective survey	Use of CT scans or the 3D model improved fracture classification as compared to standard XR; there was no significant difference between use of the CT scans and 3D models	Improve the accuracy; provide tactile feedback	Highly costly
Wu et al. (2018)	Four kinds of fracture (spinal fracture, pelvic fracture, upper limb fracture, lower limb fracture)	90 medical students, divided into 2 groups	Obtain the CT data, print 3D models; 2 groups: a traditional radiographic image group and a 3D printed model group	Test and analog scale of satisfaction	3D printed model group do better and faster in the upper limb or lower limb test, but no significant differences were found in the upper limb or lower limb test	Improve students' understanding of anatomy and fracture in complex sites	No advantage in the upper limb or lower limb; the process takes time
Huang et al. (2018)	Acetabular fracture	141 medical students	Randomly divided into 3 groups physical model (PM) group VR group and 3DP models group	Three-level objective test and subjective questions	3DP group show a clear advantage over the PM and VR group; 3DP was considered as the most valuable	Provide tactile feedback; effective in learning; promote subjective interest; improve fracture classification	No published
Li et al. (2015)	Spinal fracture	120 medical students, divided into 3 groups	Randomized into three teaching module groups (CT, 3D, or 3Dp)	Objective test and subjective questions	Students in 3DP group answer the questions better and faster	Improve the identification of complex spinal fracture anatomy	Lengthy printing time
Cao et al. (2017)	Complex fractures	80 undergraduate intern students in Grade 5, divided into 2 groups	3D printing technology to print out the complex fracture model in the experimental group; the traditional teaching method in the control group.	The understanding scores of the fracture, the judgment of preoperative and postoperative fracture consistency, the representation of fracture type, the operation ability.	The experimental group's scores were higher than the control group's, and the differences were statistically significant (P <0.05).	Let students understand the fracture situation more directly, and can observe and touch the fracture model in three dimensions	No published
Meng et al. (2018)	Complex articular fracture	60 standardized training trainees of Shanghai resident physicians in bone trauma rotation in changzheng hospital, divided into 2 groups	The control group adopted the simple conventional teaching method, and the experimental group conducted clinical teaching combined with 3D printed specimens on this basis.	To compare the basic data and test scores of the two groups.	The theoretical assessment score of the test group was, higher than that of the control group, the difference was statistically significant (*P* < 0.05).	Provide spatial details and tactile feedback.	It takes a long time, costs a lot and the demand for machines is high.
Chuang et al. (2017)	Limbs fracture	The undergraduate students in 2017, randomly divided into 2 groups, 30 in each group.	Traditional teaching In the control group. Traditional teaching methods and the 3D printing model in the experimental group.	Conduct a questionnaire survey to evaluate the teaching effect.	In terms of clinical skills, the scores of the experimental group were significantly higher than those of the control group (P <0.05).	Makes teaching more vivid, three dimensional and image, making medical students easier to master Department of orthopedics knowledge.	No published
Tan et al. (2019)	Condylar fracture	50 undergraduates majoring in stomatology general standardized training doctors, divided into 2 groups	Traditional teaching methods in the control group; the new teaching model of combination 3D printing of condylar fracture in the experimental group.	Assessed by theory and skill, and the learning effect was evaluated by questionnaire.	The clinical skill assessment of the experimental group was significantly higher than that of the control group.	Improve the interest in learning, greatly improve the concentration in class, and firmly grasp the basic theoretical knowledge	Lengthy printing time

## Discussion

Understanding the knowledge of fractures, especially of the complex structures, appropriately is a fundamental educational component but a difficult point for medical students and residents. Several complex parts of bony anatomy structure such as acetabulum and spine cause it hard for students learning with only 2D images to fully understand the morphological characteristics of relative fracture or create three-dimensional concepts in their mind. And cadaveric specimens are scarce and usually normal types without fracture for students to study (Liu et al., 2020). Similarly, the conventional physical models which can be mass-produced are also normal types. So, there are several limitations in conventional teaching methods of fracture teaching and learning.

Three-dimensional (3D) printing is a potential solution for those problems, with the ability to build specified and highly accurate 3D models quickly (Baskaran, 2016; Garcia et al., 2018) Therefore, 3D printing has special advantages in fracture-associated education and training. Its use in medical education is gradually becoming a hot spot in recent years and there have been many pieces of research in this field already. However, rigorous experimental studies are still scarce in this area, most studies remain subjective and qualitative. Teachers can prepare patient-specific models according to their needs, making the procedure of teaching more flexible and diverse.

All of the eight publications we reviewed had demonstrated that 3D printing improves the learning outcomes in education of one kind or several kinds of fractures significantly, for medical students or residents. Compared to the conventional teaching method, the most significant benefit of using 3D printed models is that it is closest to the real situation. From this perspective, it can be seen as the substitute for cadaveric specimens, which are too precious and rare to use for training usually. Lim et al. (2018) used 15 station testing to objectively evaluate the influence of XR, CT, and 3D printing on residents' judgment of acetabulum fracture classification and conducted a questionnaire survey of residents participating in the experiment. Interestingly, in the questionnaire survey, there are slightly more people who think that 3D printing is sufficient to help determine the type of fracture than those who think that CT is enough to help determine the type of fracture, but in the objective testing results, there was no significant difference in the odds ratio between CT vs. 3D models groups. At the same time, it is generally believed among the respondents that 3D printing has increased their confidence in identifying the type of fracture. This may be a psychological effect that participants think 3D printing can bring some improvement to the recognition success because the 3D model is closer to the real situation. The classification of acetabular fractures using CT or 3D printing has similar effects. Certainly, the residents may be more exposed to CT during previous learning and training. Perhaps with the same experience of using CT or 3D models, 3D printing will indeed improve the recognition success, which needs further experimental verification. The research of Wu et al. (2018) has given us a major inspiration that 3D printing has different effects on the teaching of anatomy in different parts. In some simple parts such as the upper and lower limbs, the two-dimensional teaching with CT is enough to meet the needs of undergraduates. 3D printing did not bring significant improvement. But for more complex parts such as the pelvis and spine, 3D printing models were demonstrated to make sense in improving teaching effects. The higher scores and shorter completion time tell us that 3D printing has brought undergraduates a better understanding of complex bones. Therefore, in the future, similar larger-scale and systematic teaching experiments on more parts should be conducted to make clear which part of the teaching can be improved by 3D printing. The multi-center research conducted by Huang et al. (2018) has high credibility. The authors have compared the teaching effects of ordinary models, virtual reality (VR) virtual models, and 3D models. The objective results show that 3D models and VR models are superior to ordinary models in many aspects. And 3D is the best choice among the three in more aspects; subjective survey results show that students have more interest in learning with 3D printing and VR models. Therefore, 3D printed model is considered the most valuable learning material for understanding acetabular fractures. VR is similar to 3D printing in terms of visualization but does not have the characteristics of 3D printing in terms of tangibility, this may cause the difference between the two leading methods. Similar research has been conducted by Li et al. (2015) who Compare the teaching effect of CT, 3D image, and 3D model, using subjective and objective assessment methods. Both two aspects of the assessment show that both 3D images and 3D models are better choices. Interestingly, although there is no difference in the objective outcomes in the 3D image and 3D group, in the 3D image group, males perform better than females, which did not appear in the 3D group. In addition to showing that men may be better than women in spatial awareness, this result also tells us that 3D models are more suitable for application and teaching to improve the performance of both men and women as much as possible is a better choice when compared to VR or 3D images.

We noticed that all of the subjective survey outcomes are very positive, telling us 3D printing brings confidence and interest in a relative educational procedure to the students, which is a promoter for teaching and learning. 3D printing not only prevails other teaching methods objectively but also plays a very positive role in changing the subjective attitude of students to learning. In a word, 3D printing can be regarded as the most ideal teaching aid among available ones now.

## Conclusion

During the scientometric assessment and the extensive literature analysis, we noticed the great power of 3D printing using in fracture-associated medical education. It reveals the application of 3D printing for fracture education and we can realize that it has an excellent promotion effect for medical beginners to learn fractures, especially the complex sites. Compared to traditional teaching methods, 3D printing affords better tactile feedback, which is the reason why 3D printing is more popular with students. Besides, in the subjective questionnaire survey, it was found that 3D printing has a higher mean score of satisfaction. Therefore, we believe that 3D printing models should be more widely used in the basic teaching of fractures.

However, when learning normal bone structure, ordinary bone models are sufficient for daily teaching, and there is no need to use relatively expensive 3D printing. As for the relatively less complex bone sites (upper limb and lower limb), the teaching effect brought by 3D printing is not so significant, using 2D CT images (or 3D reconstruction images is enough to achieve high-level teaching effects at low cost. Furthermore, what kind of material is used for 3D printing is also exceedingly important. Today the printing materials are mainly biological, organic, and inorganic materials. The current 3D printing model can replicate the original shape perfectly, but still needs improvement in terms of feel and strength. Using the 3D printed models, we may get better tactile feedback in the future.

We hope that in the future, there will be much more relative rigorous educational trials conducted to make clear the feasibility and necessity of using 3D printing in various teaching scenarios and that 3D printing continues to further improve clinic performance of doctors and medical education from several perspectives.

## Data Availability Statement

The original contributions presented in the study are included in the article/supplementary material, further inquiries can be directed to the corresponding author/s.

## Author Contributions

JS and SX collected the literature and drafted the initial manuscript. SF assisted in the preparation of the figures and table. MC revised the manuscript and edited the language. MZ was the lead investigator. JS, SF, MC, SX, and MZ contributed to the study's conception and design. All authors approved the final manuscript as submitted and are accountable for all aspects of the work.

## Funding

This work was supported by the Excellent Postdoctoral Program for Innovative Talent of Hunan (2020RC2015), the China Postdoctoral Science Foundation (2020TQ0364) and the Natural Science Foundation of Hunan (2020JJ5865).

## Conflict of Interest

The authors declare that the research was conducted in the absence of any commercial or financial relationships that could be construed as a potential conflict of interest.

## Publisher's Note

All claims expressed in this article are solely those of the authors and do not necessarily represent those of their affiliated organizations, or those of the publisher, the editors and the reviewers. Any product that may be evaluated in this article, or claim that may be made by its manufacturer, is not guaranteed or endorsed by the publisher.
